# Molecular complexity of the differential growth of freshwater diatoms along pH gradients

**DOI:** 10.1093/ismeco/ycaf078

**Published:** 2025-05-06

**Authors:** Xènia Rodríguez-Miret, Marisol Felip, Eric Pelletier, Jordi Catalan

**Affiliations:** Centre de Recerca Ecològica i Aplicacions Forestals, CREAF, 08193 Cerdanyola del Vallès, Spain; Departament de Biologia Evolutiva, Ecologia i Ciències Ambientals, Universitat de Barcelona, UB, 08028 Barcelona, Spain; Centre de Recerca Ecològica i Aplicacions Forestals, CREAF, 08193 Cerdanyola del Vallès, Spain; Génomique Métabolique, Genoscope, Institut de Biologie François Jacob, CEA, CNRS, Univ. Evry, Université Paris-Saclay, 91000 Evry, France; Research Federation for the Study of Global Ocean Systems Ecology and Evolution, FR2022/Tara Oceans GO-SEE, CNRS, 75016 Paris, France; Consejo Superior de Investigaciones Científicas, CSIC, 08193 Cerdanyola del Vallès, Spain; Centre de Recerca Ecològica i Aplicacions Forestals, CREAF, 08193 Cerdanyola del Vallès, Spain

**Keywords:** transcriptomics, environmental change, adaptation, acidic environments, niche, evolution, algae, cell biology

## Abstract

Diatoms originated in marine waters, and many clades have invaded fresh waters, radiating throughout the continental aquatic environments. pH gradients have been a primary axis of species differentiation, from which environmental assessments have taken advantage using diatoms as bioindicators. However, a comprehensive view of the molecular variation underlying the diatom sensitivity to pH is missing. This study used 12 freshwater diatom strains across a broad phylogenetic range within raphid pennate clades and 3 distinct environmental pH conditions, pH 4.7, 7.0, and 8.2, for a common garden experiment. The transcriptomic analysis showed that environmental pH variation regulated many molecular processes and biological functions, especially those involved in biosynthesis and transport. Despite sharing many known functions, strains responded to pH changes in a highly idiosyncratic manner. Such specificity in the physiological response to pH aligns with the considerably divergent genetic backgrounds observed among the 12 diatom strains. This variation was likely shaped by different evolutionary trajectories in adaptive molecular landscapes, which were probably already differentiated in the initial marine environment and subjected to varying pH selection pressures in the complex chemical mosaic of inland waters. Overall, our results indicate that continental pH selection pressures do not determine a necessarily unique adaptive physiological response in diatoms, but instead allow for multiple adaptive solutions built on the evolutionary historical background and inland contingencies. Therefore, specific studies on the identified plastic responses to pH are needed to assess their adaptive function across clades.

## Graphical Abstract

**Graphical Abstract:**
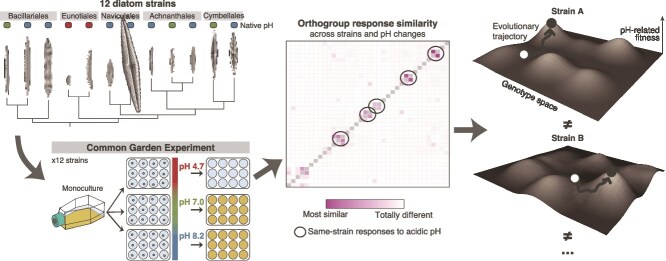

## Introduction

In a world where ongoing climate change and anthropogenic activities substantially impact aquatic ecosystems, freshwater diatoms are widely used as indicators of ecological and environmental conditions [1, 2]. Diatom communities show a high sensitivity to pH [3, 4], with their relative composition able to respond within a few days to pH fluctuations [5, 6]. Consequently, many studies have determined the pH optima and tolerance of numerous diatom species for environmental applications [7–10]. However, there is a large knowledge gap regarding the genetic and molecular mechanisms underlying the sensitivity of diatoms to pH and the resulting species segregation along the pH gradients. Some adaptations of acidophilic and alkalophilic algae to their extreme environments have been described [11, 12]. However, diatoms possess a unique combination of cellular traits that could have resulted in substantial differences in their eco-evolutionary trajectories compared to other groups. These traits include a silica cell wall called the frustule [13, 14], complex chloroplasts with four membranes derived from two successive endosymbiotic events [15], a highly adaptive and efficient photosynthetic apparatus [16, 17], a complete and functional ornithine–urea cycle similar to that in metazoans [18, 19], and a considerable number of horizontally transferred genes acquired (especially from bacteria) throughout diatom evolution [20, 21].

Diatoms originated in the marine environment, and successive invasions of continental waters by the main clades resulted in higher speciation rates than in the sea [22]. Given the buffered pH conditions in the ocean, continental pH gradients are likely among the primary drivers of evolutionary change, as indicated by the species segregation across them. The remarkable difference between the initial marine pH and some extremely acidic continental waters may have imposed similar physiological challenges to all clades that have succeeded in invading low pH habitats [23, 24]. Because selection works on undirected variation, there is much potential for chance to have played a role in the evolutionary solutions each clade developed to adapt to these extreme external conditions. However, the historical evolutionary background could have constrained these solutions, particularly because specific clades invaded inland waters from the sea at different periods over millions of years. Alternatively, an intense selection pressure due to highly specific and strong physiological constraints of the low environmental pH might have resulted in unique, necessary, adaptive convergent molecular traits. This study aimed to evaluate these three components, chance, history, and necessity, on the colonization of freshwater pH gradients by diatoms, comparing the molecular responses of phylogenetically diverse diatom strains from lakes of contrasting pH using a common garden experiment and transcriptomics.

## Materials and methods

### Sample collection and single-cell isolation

To obtain diatom species adapted to different environmental conditions, we sampled four Pyrenean lakes with distinct pH and alkalinity conditions: the acidic Lake Aixeus (pH 4.89), the pH-neutral Lake Redon (pH 6.97) and Lake Redó (pH 7.04), and the alkaline Lake Estanya (pH 8.11). The four lakes are located in the southern central part of the Pyrenean mountain range. In each lake, water samples for pH measurement were collected, and epilithic biofilms were scraped from 15 to 20 submerged littoral rocks (focusing on the top surfaces) using a clean brush and then placed into sterile tubes.

Twelve strains from different species were isolated from the collected biofilm samples by single-cell pipetting and suction using capillary pipettes under an inverted microscope. Monocultures were grown using three distinct growth media (Supplementary Table S1) based on nutrient concentrations and adjusted pH: WC medium [25] adjusted to pH 7.0 (WC7) was used for diatoms isolated from Redó and Redon samples, WC medium adjusted to pH 8.2 (WC8) for diatoms isolated from Estanya, and PM medium [26] adjusted to pH 4.7 (PM4) for diatoms isolated from Aixeus.

Slide preparation for taxonomic identification of the strains was performed following Battarbee et al. [27]. All obtained monocultures were pennate diatoms from the Bacillariophyceae class belonging to five different orders: Bacillariales, Eunotiales, Naviculales, Achnanthales, and Cymbellales (Supplementary Fig. S1; Supplementary Table S2). Eunotiales, Naviculales, and Achnanthales were each represented by a single genus, whereas Bacillariales and Cymbellales included species from two different genera. The 12 strains were named using a code for the species name and the strain number from our lab culture collection.

### Common garden experiment

Common garden experiments (CGEs) consist of growing genetically distinct taxa under the same environmental conditions. This study used 12 diatom strains and 3 distinct environmental pH conditions: pH 4.7, 7.0, and 8.2 (Supplementary Fig. S2). Each strain and pH condition combination was cultured in a separate 12-well plate. The experimental culture plates were kept at 15°C with a 12:12 light–dark cycle and an irradiance of ∼90 μmol photons m^−2^ s^−1^ throughout the experiment. The experimental cultures were renewed every 3 days (except for the 4-day acclimatization period) by medium renewal or reinoculation to keep the populations in exponential growth and maintain the original experimental environmental parameters. The growth rate of the experimental populations was assessed by fluorescence monitoring (using excitation and emission wavelengths of 440 nm and 680 nm, respectively [28]) during distinct consecutive growth periods, defined as the intervals between culture renewals. The dark-adapted minimal fluorescence (*F*_0_) parameter has been identified as a reliable fluorescence-based biomass proxy for benthic diatoms [29]. To measure *F*_0_, the experimental culture plates were kept in the dark for 15 min before the fluorescence measurement. A single fluorescence measurement was obtained from the central top of each well with the Varioskan LUX multimode microplate reader. For each well and growth period, the growth rate (*μ*) was calculated as:


$$ \mu =\frac{\ln \left({N}_1\right)-\ln \left({N}_0\right)}{t_1-{t}_0} $$

where *N*_0_ represents relative fluorescence units (RFU) at the beginning of the growth period (*t*_0_) and *N*_1_ represents RFU at the end of the growth period (*t*_1_). Only the two growth periods after growth rate stabilization (acclimatization period) were selected for subsequent analysis except for rapidly decaying samples, for which only one period could be selected. Kruskal–Wallis and post-hoc Dunn’s tests were conducted for each strain to determine which experimental pH conditions showed significantly different growth rate medians. The analyses were conducted using R 4.3.1 [30]. Kruskal–Wallis tests were performed using the kruskal.test function (with default settings). Dunn’s tests were performed using the DunnTest function (with default settings and method = “fdr”) from the DescTools R package [31]. The false discovery rate (FDR) threshold used for significance was .05 for both Kruskal–Wallis and Dunn’s tests.

After at least 10 days of experimental culture growth since the first inoculation, the biofilm from the wells was completely scraped off, and total RNA was extracted and purified using the Direct-zol RNA Miniprep Kit (Zymo Research). Three RNA replicates were obtained per plate (i.e. for each strain and pH condition) by pooling samples from four wells (Supplementary Fig. S2). Total RNA was quantified using the Qubit fluorometer with the Qubit RNA High Sensitivity (HS) Assay kit. In total, 80 RNA samples were obtained (Supplementary Table S3).

mRNA enrichment, library preparation, and sequencing were performed by Novogene Company Ltd. (UK) following their standard protocols. Magnetic beads coated with poly-T oligos were used to selectively capture mRNA from the total RNA sample. The library was then constructed. The quality of the library was assessed using Qubit and real-time PCR for quantification and Bioanalyzer for size distribution detection. Finally, libraries were pooled and sequenced on Illumina NovaSeq PE150. Reads were filtered by the Illumina quality filters.

### Bioinformatics workflow

The bioinformatics workflow comprised several phases (Supplementary Fig. S3). The raw reads were processed into clean, non-ribosomal reads by Genoscope (France) using its custom filtering and quality control methods, detailed in Alberti et al. [32]. For each strain, clean reads from all samples were used as input for the *de novo* transcriptome assembly performed with Trinity 2.11.0 [33] (with default settings). Transcripts were examined using BLAST+ 2.12.0 [34] to identify potential bacterial contamination. Transcripts with bacterial hits (with default settings and E-value ≤10^*−*20^, percent identity *≥*90%, percent query coverage per hsp ≥ 40%) and no Ochrophyta hit based on either blastn or blastx results were removed and hence not included in subsequent analyses. The summary of general transcriptome assembly metrics was obtained using the TrinityStats.pl Trinity script.

Proteins were predicted from transcripts using TransDecoder 5.5.0 scripts (https://transdecoder.github.io/). First, open reading frames (ORFs) on the top strand containing at least 100 amino acids were retrieved using the TransDecoder.LongOrfs script (with default settings and -S). Then, two searches on the predicted ORFs for homology to known proteins were performed: a blastp search against the Swissprot protein database using BLAST+ 2.12.0 [34] (with default settings and -max_target_seqs 1, -evalue 1e-5) and a protein domain search with Pfam 35.0 [35] and HMMER 3.3.2 [36] (with default settings and -E 1e-10). These two homology searches were included as ORF retention criteria in the second TransDecoder script used, TransDecoder.Predict (with default settings). For transcripts with no retained protein based on homology, their longest ORF was kept as a predicted protein. The longest ORF per transcript was retrieved using the get_longest_ORF_per_transcript.pl script from TransDecoder.

Predicted protein sequences were annotated with InterPro [37] and Gene Ontology [38, 39] terms using InterProScan 5.61.93.0 [40]. Parent terms of the Gene Ontology terms retrieved by InterProScan were also assigned to each sequence using the hierarchy among terms from the R package GO.db 3.17.0 [41]. Predicted proteins were also annotated with KEGG Orthology (KO), KEGG MODULE, and KEGG BRITE terms from the KEGG database [42, 43] using KofamScan 1.3.0 [44]. The prediction of protein location was obtained from TargetP 2.0 [45] with the “non-plant” organism group selected (default settings and -org non-pl) and ASAFind 2 [46] (with default settings and -ppc), since this combination is suitable for diatoms [47]. The number of transmembrane helices within each protein sequence was predicted using TMHMM 2.0 [48]. The inference of phylogenetic relationships among the proteins from the 12 strains was performed with OrthoFinder 2.5.4 [49, 50].

Differential expression (DE) analyses were conducted independently for each strain using Trinity 2.11.0 utilities [33]. First, the align and estimate abundance.pl Trinity script (with default settings and est_method RSEM, aln_method bowtie2, SS_lib_type FR, prep_reference) was used for the alignment-based quantification of genes. This utility required RSEM 1.3.3 [51]. Gene expression matrices were created with the abundance_estimates_to_matrix.pl Trinity script (with default settings and est_method RSEM). A DE analysis for genes was performed for each pairwise pH comparison using the run_DE_analysis.pl Trinity script (with default settings and method edgeR), which required edgeR software package [52]. Only genes with ≥1 counts per million (CPMs) in at least two replicates (default setting for --min_reps_min_cpm) were included in the analyses. Then, the analyze_diff_expr.pl Trinity script (with default settings and -P 0.01, -C 0) was employed to extract differentially expressed genes (DEGs) for each pairwise pH comparison, with an FDR cut-off of .01 for significance. This script also provided Pearson correlation coefficients and Euclidean distances among samples based on centered log2-transformed gene expression values. When referring to DE (and enrichment patterns, described below), the expression at the lowest pH is compared to that at the highest pH; e.g. a downregulation in the pH 4.7 vs. 8.2 comparison means that the expression was significantly lower at pH 4.7.

Enrichment analyses were performed for each strain and pairwise pH comparison to detect patterns of gene DE (i.e. enrichment or depletion) in specific gene sets. Six gene set types were included, namely InterPro, Gene Ontology, KEGG KO, KEGG BRITE, and KEGG MODULE terms, and orthogroups. Three complementary methods for enrichment analysis were used (for a description of each method, see below). A gene set was considered enriched when the enrichment was significant in at least one of the three methods, and the same for depletions. Affected gene sets were classified into strain-pH enrichment groups, defined by their enrichment direction (enriched or depleted) in a specific pairwise pH comparison and strain. These groups were named using the strain name followed by the enrichment direction (En, enriched; Dp, depleted) placed between the two pH conditions being compared (for simplicity, pH values were truncated to 4, 7, and 8); e.g. the group “NPAL-12_7En8” contains gene sets enriched at pH 7.0 compared to pH 8.2 in strain NPAL-12. The dissimilarities among strain-pH enrichment groups were computed using the Euclidean-like, square-root-transformed Jaccard distance [53, 54], based on the presence (1) or absence (0) of the gene sets within each group. The distances were obtained with the dist.binary function from the ade4 R package [55]. All analyses were conducted using R 4.3.1 [30].

Two of the three methods used for enrichment analysis belong to two widely used gene set analysis (GSA) categories: functional class scoring (FCS) and over-representation analysis (ORA) [56–58]. The FCS analysis identified gene sets with an overall tendency of gene upregulation (enriched) or downregulation (depleted). For this analysis, gene-level scores were calculated as signed ln(FDR) (SLFDR) values, derived from the FDR and fold change (FC) values of the DE analysis using the following formula:


$$ \mathrm{SLFDR}=\ln \left(\mathrm{FDR}\right)\cdotp \frac{\log_2\left(\mathrm{FC}\right)}{\left|{\log}_2\left(\mathrm{FC}\right)\right|} $$

Then, for each gene set, the gene-level scores from genes within and outside the set were aggregated into a global statistic reflecting the overall expression trend within the set. The statistical significance of each global statistic was assessed using the non-parametric Wilcoxon rank-sum test [59] based on gene sampling. This analysis was performed with the runGSA function of the R package piano [60]. The ORA analysis detected gene sets containing a significant number of upregulated genes (enriched), downregulated genes (depleted), or both. In this analysis, a chi-square test was applied to each gene set to statistically determine whether the proportions of significantly upregulated and downregulated genes within the set were higher than those outside it. The chi-square test was computed using the chisq.test function from the R package stats. For both FCS and ORA, an FDR cut-off of .01 was used for significance. The third enrichment analysis is a new method named the unidirectional gene set (UGS) analysis. The UGS focused on unidirectional responses by identifying gene sets in which all DEGs were either exclusively upregulated (enriched) or downregulated (depleted), regardless of their proportion within the gene set.

## Results

### Growth patterns along the pH gradient

**Figure 1:**
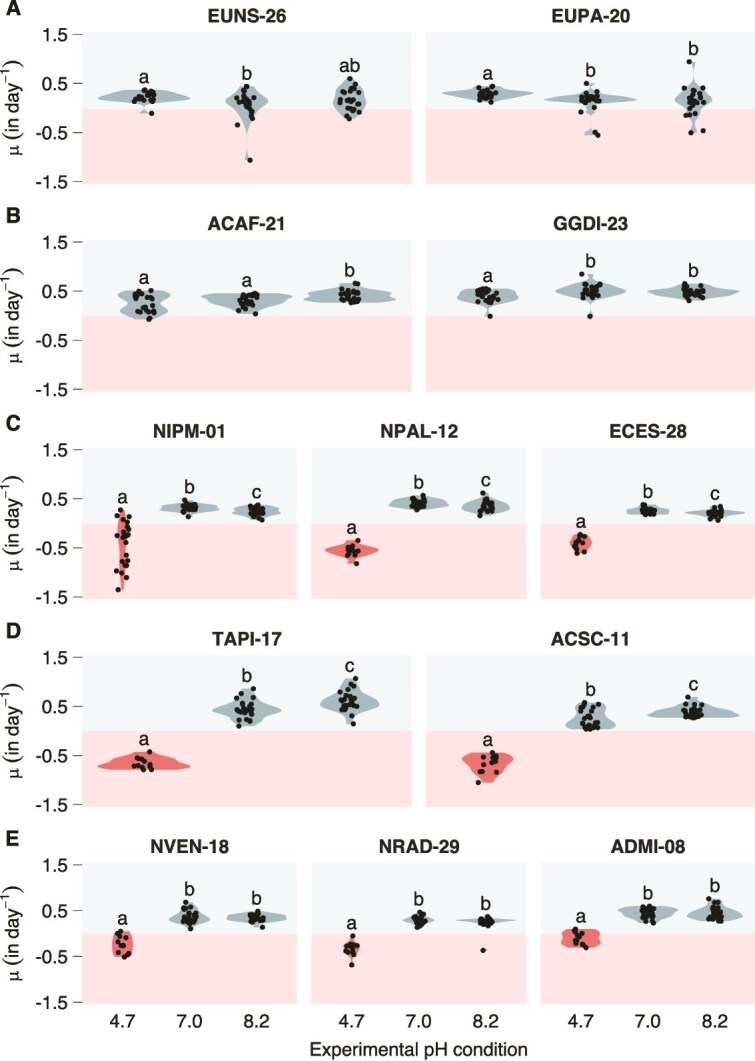
Growth rate at experimental pH 4.7, 7.0, and 8.2 for the 12 diatom strains. The strains are grouped into acidophiles (A), generalists (B), and acid-intolerants with higher (C), lower (D), or similar (E) growth at pH 7.0 compared to pH 8.2. Blue and red violins represent positive and negative median growth rates, respectively. Compact letter display on top of the violins identifies statistically indistinguishable groups (Dunn’s FDR threshold = .05) within each strain. *N* = 24 for all growing populations and NIPM-01 decaying populations, and *N* = 12 for the remaining decaying populations.

The 12 diatom strains studied in this investigation were grouped into 3 main growth patterns across the pH gradient (Fig. 1; Supplementary Table S4). *Eunotia* EUNS-26 and EUPA-20 from Lake Aixeus were characterized as acidophiles because their fastest growth occurred at pH 4.7, although they showed slow but positive growth under neutral and alkaline conditions. *Achnanthidium* ACAF-21 and *Gomphonema* GGDI-23 from Lake Redon were characterized as generalists because they showed considerable growth under all three experimental pH conditions. Even though acidophiles and generalists showed their best and worst growth, respectively, at pH 4.7, the median growth rate was not significantly different between the two groups under this pH condition (Dunn’s FDR = .168). The remaining eight strains, isolated from Lake Redó and Lake Estanya, were characterized as acid-intolerants because their populations collapsed at pH 4.7 a few days after inoculation. Some acid-intolerant strains showed slight but significant differences between growth at pH 7.0 and 8.2, with some growing faster at alkaline pH and others at neutral pH.

### Transcriptome characteristics

**Figure 2:**
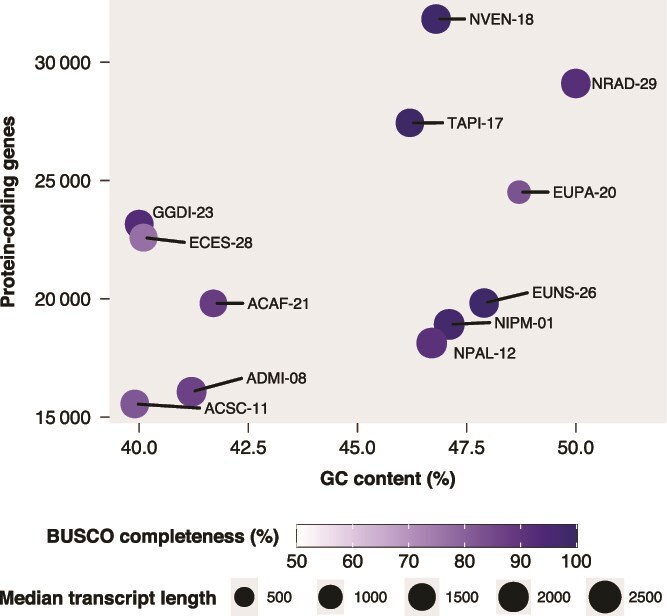
Summary of the 12 transcriptome assemblies. BUSCO completeness includes both complete and fragmented BUSCO sequences. Median transcript length was determined using the longest transcript sequence of each gene.

Total detected genes in the protein-coding transcriptomes ranged from 18 699 in *Achnanthidium* ACSC-11 to 43 521 in *Navicula* NVEN-18, which could be the diatom with the largest protein-coding transcriptome sequenced to date. A relatively large proportion of genes showed low expression in each strain. Henceforth, when referring to genes and their predicted proteins, we will consider the subset of genes and transcripts with expression above the established minimum threshold (i.e. with CPM ≥ 1 in at least two replicates). Considering this, the number of protein-coding genes ranged from 15 556 in ACSC-11 to 31 827 in NVEN-18 (Fig. 2; Supplementary Table S5). Two *Achnanthidium*, ACSC-11 and ADMI-08, were the strains with the lowest number of protein-coding genes and transcripts detected. In contrast, the two *Navicula*, NVEN-18 and NRAD-29, and *Tryblionella* TAPI-17 showed the largest number of protein-coding genes. The mean number of transcripts per gene for each strain generally spanned from 1.8 to 2.2, with at least 42.7% of genes in each strain producing a single transcript. Exceptionally, in EUPA-20, the mean number of transcripts per gene was 1.4, with 75.4% of genes encoding a single transcript, whereas in NIPM-01, the number of transcripts per gene was 2.6 on average, and 34.3% of genes produced a single transcript. EUPA-20 also showed the smallest median transcript length for the longest transcript per gene, but this could be artefactual according to the considerable proportion of partial proteins in this strain. The GC content in protein-coding genes varied among phylogenetic clades, being lower in the three *Achnanthidium* and the two Cymbellales strains. Based on the predicted protein dataset, the 12 protein-coding transcriptomes exhibited considerable completeness, with at least 77 of the 100 stramenopile BUSCOs found (considering both complete and fragmented). A large proportion of the detected BUSCOs were complete and not fragmented.

**Figure 3:**
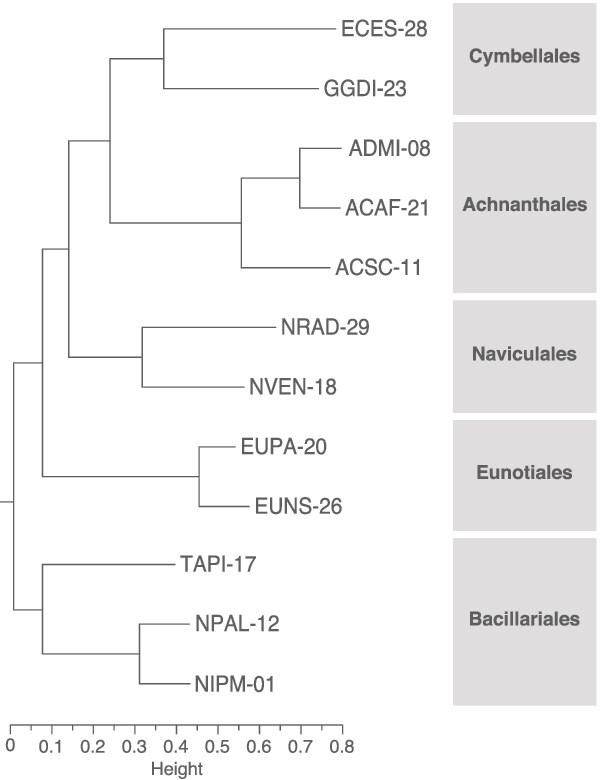
Phylogenetic tree of the 12 diatom strains. The tree was inferred from gene trees of shared orthogroups using OrthoFinder and was manually rooted to be consistent with the Nakov et al. [61] tree.

**Figure 4:**
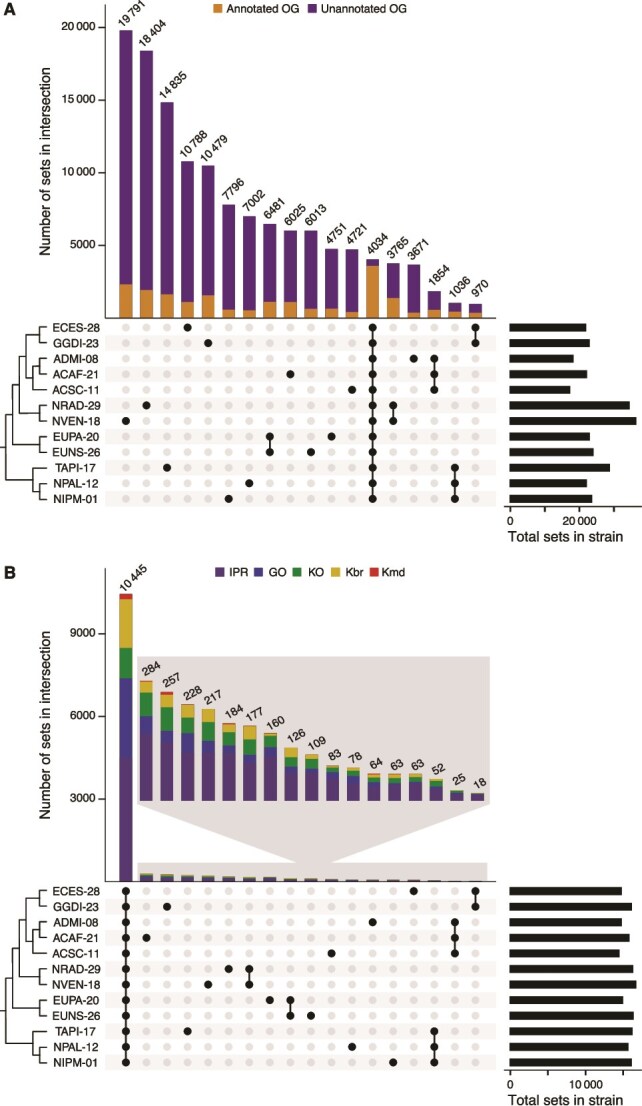
Number of orthogroups (A) and functional sets (B) shared across the 12 diatom strains. The vertical bars in the UpSet plot represent the number of orthogroups (A) and functional sets (B) found exclusively in all strains of the intersection indicated in the matrix below. Not all strain combinations are displayed. The total number of orthogroups (A) and functional sets (B) in each strain is represented by horizontal bars to the right of the matrix. A cladogram illustrating the phylogenetic relationships among species (corresponding to the dendrogram in Fig. 3) is shown to the left of the matrix. OG, orthogroup; IPR, InterPro; GO, Gene Ontology; KO, KEGG Orthology; Kbr, KEGG BRITE; Kmd, KEGG MODULE.

Predicted proteins were assigned to orthogroups based on sequence homology. The phylogenetic tree obtained for the 12 strains was consistent with their taxonomic classification (Fig. 3; Supplementary Table S2). In total, 4034 orthogroups were present in all 12 strains, representing between 11.1% and 23.6% of total orthogroups per strain (Fig. 4A; Supplementary Table S5). At the strain level, the percentage of strain-specific orthogroups was mainly higher than both common and order-specific orthogroups, representing at least 20.3% and up to 54.6% of total orthogroups per strain. These differences in abundance between strain-specific and both order-specific and common orthogroups were significant (Dunn’s FDR ≤ .01 in both comparisons). In contrast, the number of functional sets identified in all 12 strains was much higher than those order- or species-specific (Fig. 4B). For each functional database, the percentage of annotated proteins was similar across the 12 transcriptomes (Supplementary Table S5). In each strain, 40.0% to 50.5% of genes encoded at least one protein with annotations from the InterPro, Gene Ontology, or KEGG databases. The proportion of functionally annotated orthogroups was significantly smaller as the orthogroup age class was younger (Fig. 4A; Dunn’s FDR ≤ .01 for the three comparisons among common, order-, and strain-specific gene percentages in strains), considering an orthogroup as functionally annotated if it contained at least one annotated protein in any of the three functional databases used.

### Differential expression and enrichment analysis

When comparing the expression levels of genes, replicates from the same pH condition clustered together in all strains (Supplementary Fig. S4). In the two generalist strains, ACAF-21 and GGDI-23, gene expression at pH 4.7 was more different from both pH 7.0 and 8.2, whereas in the acidophile EUPA-20, the most dissimilar gene expression occurred at pH 7.0. Both gene upregulations and downregulations were detected in each pH comparison and strain (Supplementary Fig. S5). The balance between upregulations and downregulations varied across strains, but generally, there were no substantial differences except for *Eunotia* EUNS-26 in the pH 4.7 vs. pH 7.0 comparison.

**Figure 5:**
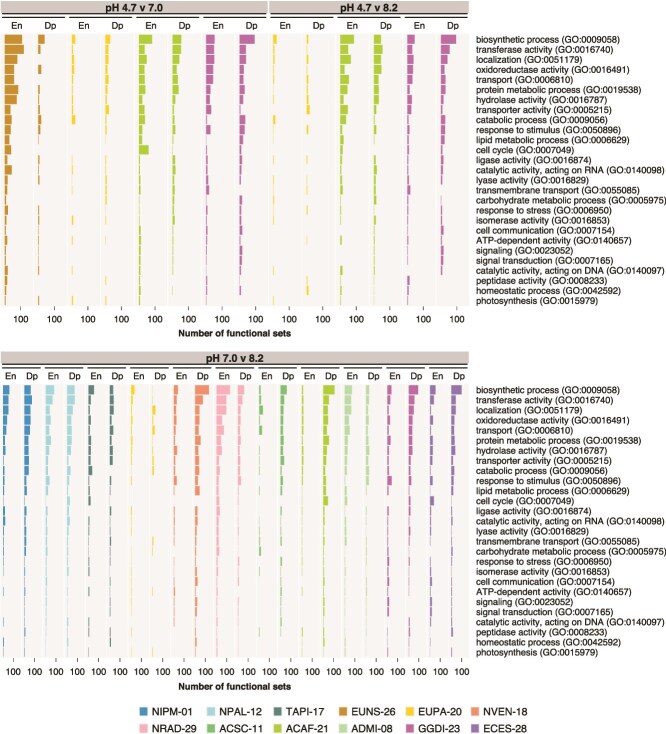
Number of enriched and depleted Gene Ontology terms per strain and pH comparison within selected general categories. Gene Ontology categories are sorted by the decreasing number of affected terms across all strains. En, enriched; Dp, depleted.

**Figure 6:**
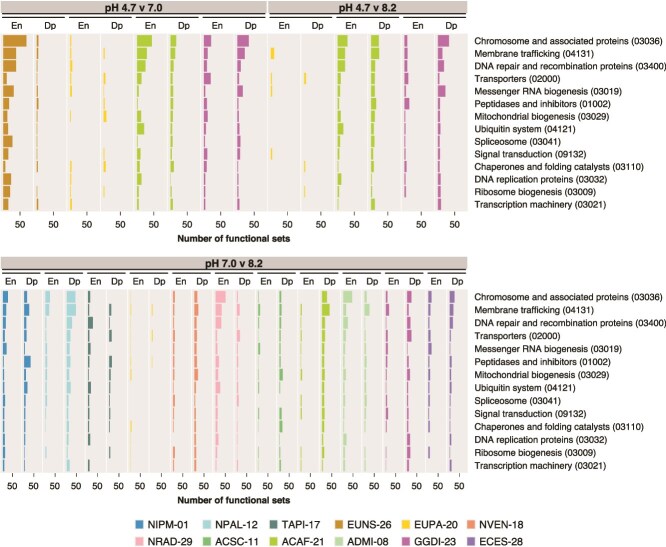
Number of enriched and depleted KEGG BRITE terms per strain and pH comparison within selected general categories. KEGG BRITE categories are sorted by the decreasing number of affected terms across all strains. En, enriched; Dp, depleted.

Based on the DE analysis, enriched and depleted gene sets were detected in all pH comparisons and strains (Supplementary Fig. S6). Many enriched and depleted Gene Ontology sets were involved in biosynthetic processes and the establishment and maintenance of cellular location (Fig. 5). Catabolism was also notably affected. Other categories with many affected Gene Ontology sets across contrasts include protein metabolism, responses to stimuli, lipid metabolic processes, cell cycle, and carbohydrate metabolic processes. Consistent with affected Gene Ontology categories, membrane trafficking, transporters, and signal transduction were among the most affected KEGG BRITE categories in all contrasts (Fig. 6). Categories associated with chromosomes and participating in DNA repair and recombination or mRNA biogenesis showed many affected KEGG BRITE sets, along with mitochondrial and ribosome biogenesis, peptidases and inhibitors, and the ubiquitin system.

**Figure 7:**
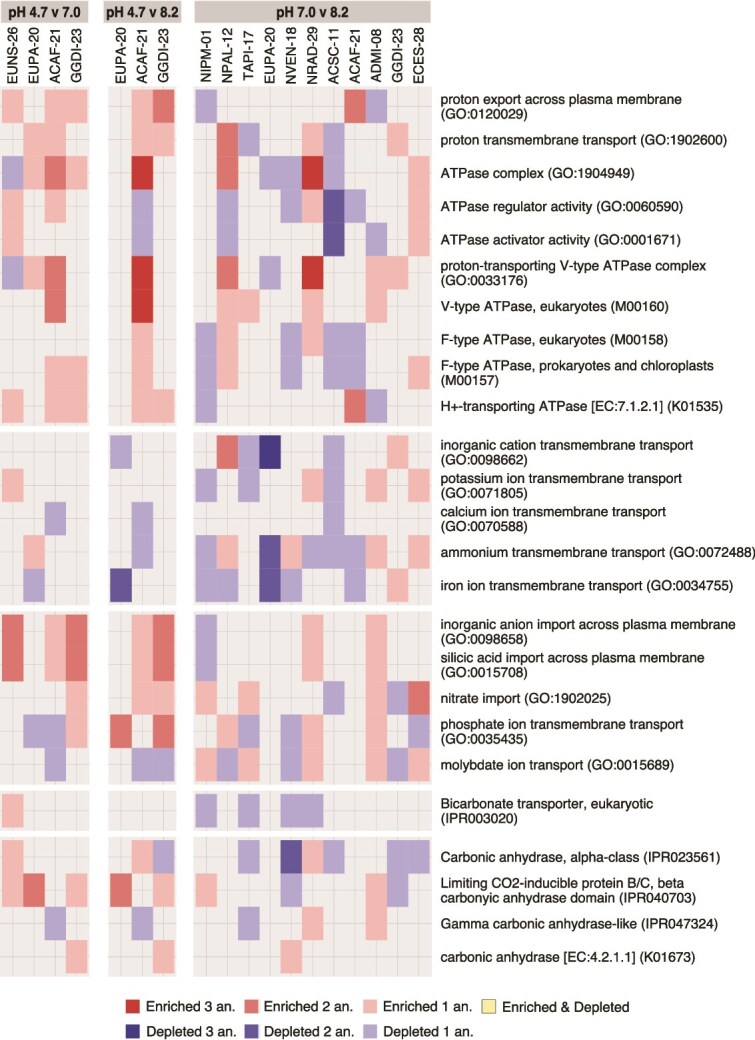
Enrichment patterns of functional sets related to pH homeostasis and transport across strains and pH comparisons. Functional sets are grouped into five categories, starting from top: proton transport, cation transport, anion transport, bicarbonate transport, and CAs. Color intensity indicates the number of significant enrichment analyses (up to three, FCS, ORA, and UGS).

Some functional sets related to pH homeostasis and ion transport were examined. These gene sets were classified into distinct functional categories: proton, bicarbonate, other cations and other anions transport, and carbonic anhydrases (CAs). The enrichment pattern of sets within each functional category generally varied across strains (Fig. 7). The transport of distinct anions and cations, including protons, was affected in many strains and pH comparisons. Both proton export by P-type ATPases and silicic acid import by silicon transporters were enriched at pH 4.7 in all acid-tolerant strains except for *Eunotia* EUPA-20. In addition, the proton-transporting V-type ATPase was also enriched at pH 4.7 in the generalist *Achnanthidium* ACAF-21. The V-type ATPase was also enriched at pH 7.0 compared to pH 8.2 in some acid-intolerant strains and the generalist *Gomphonema* GGDI-23, but depleted in *Eunotia* EUPA-20. In contrast, bicarbonate transport was depleted at pH 7.0 compared to 8.2 in four acid-intolerant strains and was enriched at pH 4.7 uniquely in *Eunotia* EUNS-26. Lastly, CAs were affected in most strains and pH comparisons, with strains exhibiting distinct combinations of affected CA types, enrichment patterns, and predicted distributions in subcellular compartments. The most common response was the enrichment of limiting CO_2_-inducible proteins B/C (LCIB/C) βCA domain-containing proteins at low pH in all acid-tolerant strains, except for *Achnanthidium* ACAF-21.

### Response clustering

**Figure 8:**
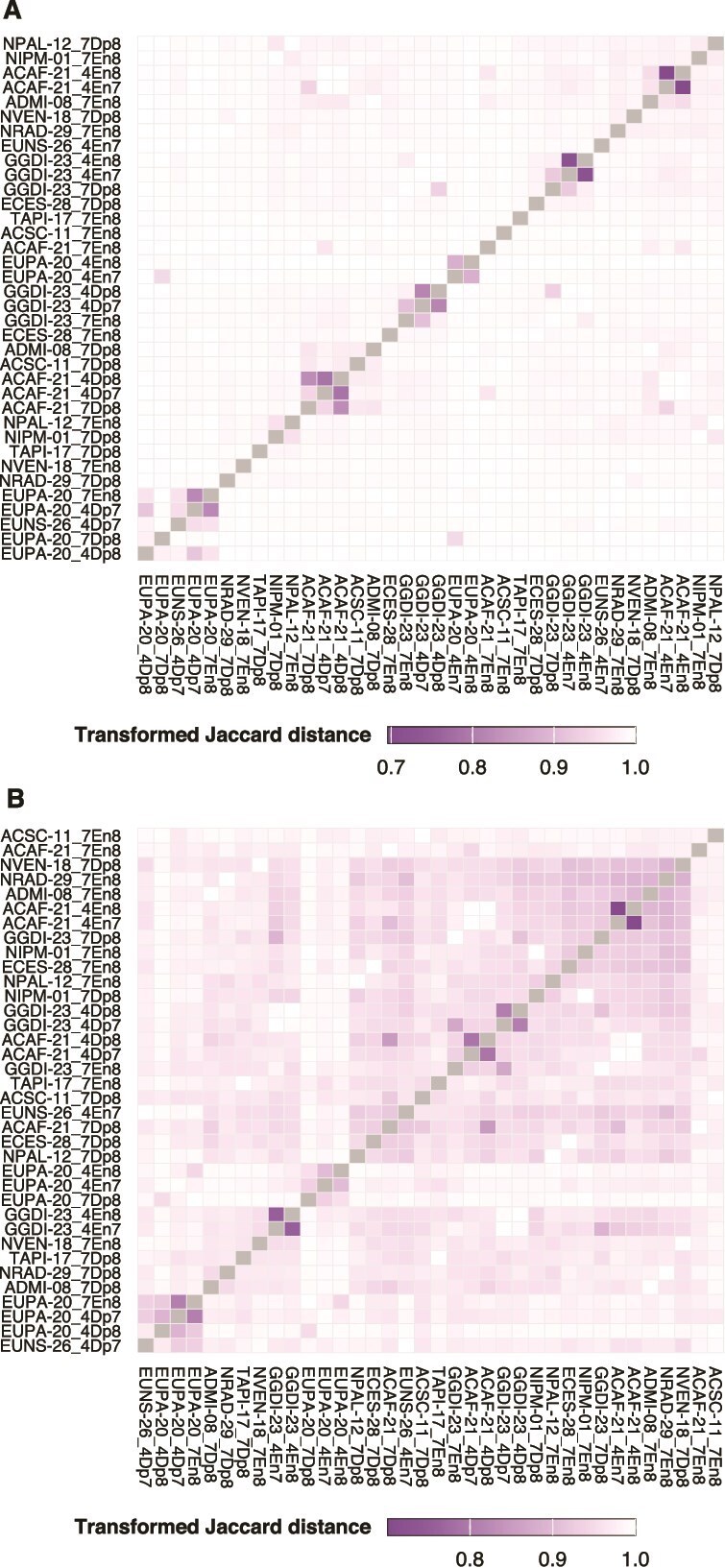
Heatmap based on orthogroups (A) and functional set (B) response similarity among strains and pH comparisons. Strain-pH enrichment groups were sorted along the axes based on dendrogram similarity (not shown). Groups were labeled using the strain name followed by the enrichment direction placed between the two pH conditions being compared (see Materials and methods). Note that, for visualization purposes, the fill gradient starts at the minimum estimated distance in each heatmap.

The affected orthogroups varied remarkably across strains and pH comparisons (Fig. 8A). However, strain-pH enrichment groups involving the same strain and enrichment direction at pH 4.7 showed a higher resemblance. The most similar responses in orthogroups were observed for enrichments at pH 4.7 compared to 7.0 and those at pH 4.7 compared to 8.2 in both ACAF-21 and GGDI-23, followed by depletions in the same pH comparisons and strains. EUPA-20 enrichments at pH 4.7 compared to 7.0 and at pH 4.7 compared to 8.2 also displayed greater similarity between them than the average. However, in EUPA-20, orthogroup depletions at pH 4.7 compared to pH 7.0 were most similar to enrichments at pH 7.0 compared to pH 8.2. Despite the low resemblance for the rest of the pairwise comparisons, phylogenetically closely related strains tended to show the most similar orthogroup responses in the pH 7.0 vs. 8.2 comparison (Supplementary Fig. S7A).

Similarly, there was little resemblance among the functional sets affected in each strain and pH comparison (Fig. 8B). However, functional responses were more similar across strains than homology-based responses. The clustering pattern of responses was similar to the results obtained for orthogroups, particularly for contrasts involving pH 4.7. On the other hand, phylogenetically closely related strains did not show a higher response similarity (Supplementary Fig. S7B).

## Discussion

This study investigated the growth and molecular responses of 12 freshwater diatom strains comparing acidic (4.7), neutral (7.0), and alkaline (8.2) environmental pH conditions. These strains represented distinct species encompassing a broad range of phylogenetic diversity within raphid pennate diatoms [61] and native pH conditions. Acidic, neutral, and alkaline pH conditions caused the regulation of a myriad of molecular functions and biological processes among the strains studied. Transcriptional change is one mechanism used to regulate the concentration of adaptive proteins and hence, the capacity for the cellular tasks these proteins facilitate [62]. As biosynthetic and physical resources are finite in cells [62, 63], the numerous and functionally diverse DEGs observed across strains and pH changes point to a wide reallocation among stress tolerance resources based on differences in niche requirements. In those pH changes in which the growth rate was affected, a proportion of these DEGs is likely directly related to changes in the growth rate rather than to stress [62, 64]. Although slight but significant phylogenetic components were identified, the diatom strains exhibited small overlap in their responses to the same pH changes, even among those showing similar growth patterns. The idiosyncratic response of strains could have derived from the interaction of the niche characteristics in which the species evolved and the presence of numerous recently emerged strain-specific genes in diatom protein-coding transcriptomes. Young genes tend to perform non-essential functions and hence are less constrained by selection than old genes, making them more likely to evolve to become specialized, niche-adaptive genes [63, 65–67]. It seems probable that differences in past evolutionary niches and genetic backgrounds among lineages translated into differences in their adaptive landscape topologies, even under the same environmental conditions. This resulted in strains following unique evolutionary trajectories and relying notably on strain-specific adaptive mechanisms for environmental tolerance [68–70]. The large proportion of functionally unannotated young genes indicates that many key niche-specialized functions in diatoms may not have been described yet.

Growth and molecular responses showed that acidic pH is a more ecologically distinct environment than neutral and alkaline pH conditions for the studied diatoms, assuming that slower (including negative) growth rates indicate higher cellular stress [62, 71]. The distinctiveness of acidic pH as an eco-evolutionary challenge is probably related to the marine ancestry of diatoms and the widespread regional distribution of circumneutral pH fresh waters across the planet [22, 72]. Our results suggest that the differential tolerance to low pH and the divergence in acid adaptations may have derived from the independent freshwater colonization events of diatom lineages, with *Eunotia*, *Achnanthidium*, and *Gomphonema* representing early freshwater colonizers from independent colonization events [22]. Marine diatoms colonizing fresh waters may require first invading fresh waters with a pH similar to marine conditions and then, allowing sufficient time for new, resource-consuming low-pH adaptations [73] to emerge randomly and be selected in some lineages within the clade. As discussed earlier, the specificity of these acid adaptations could be derived from the presence of numerous young genes. The details on the responses to acidic pH in acid-tolerant strains will be addressed elsewhere. There was apparently no relation between the number of protein-coding genes and the environmental pH tolerance of the strains. Thriving at acidic pH might involve a few genes dedicated specifically to low pH tolerance, while many proteins regulated at pH 4.7 could be involved in a core general response to stress [64, 74]. However, phylogenetic influence on the number of protein-coding genes may obscure this relationship. Within *Achnanthidium*, the generalist ACAF-21 has more protein-coding genes than the two acid-intolerant ACSC-11 and ADMI-08, aligning with a higher environmental pH tolerance associated with a higher number of protein-coding genes. Overall, although the number of strains considered is far from comprehensively covering the diatom evolutionary tree, the variety of clades considered and the complexity of growth and molecular responses along the pH gradient are sufficient to suggest that historical contingency, chance, and necessity have all played a role in adaptive evolution and species radiation across pH continental gradients [23, 24, 75].

Regarding functionality, many annotated proteins responding to pH changes were involved in activating and deactivating signaling pathways in response to stimuli and the resulting modifications in gene expression, as well as protein biosynthesis and localization. This pattern supports the idea that complex, environmentally induced expression tuning is required to meet the physiological requirements dictated by environmental conditions [64, 76]. To regulate the intracellular pH, acids or bases are actively transported across the plasma membrane [77, 78]. In this study, the higher expression of proton-transporting P-type ATPases at low pH in most acid-tolerant strains suggests that proton export is generally used by diatoms to reduce cytosolic acidification in acidic environments [11]. In contrast, its regulation seems less common between pH 7.0 and 8.2. Our results indicate that proton-transporting V-type ATPases may also participate in pH regulation at low pH through proton extrusion from the cytosol into the vacuole in some diatoms, such as *Achnanthidium* ACAF-21, and may have a greater role in neutral than in alkaline conditions in some species. Vacuolar compartmentalization has been previously described as a short-term mechanism to cope with cytosolic acidification [11, 79]. Enhanced bicarbonate transport was identified as potentially relevant for adaptation to pH 8.2 conditions, but only in some lineages. Increasing cytosolic bicarbonate concentrations raises the pH [80], which appears counterintuitive under environmental alkaline conditions, given that these conditions already promote cytosolic alkalinization [12]. However, the higher expression of bicarbonate transporters may respond to a higher need for bicarbonate transport within diatom carbon-concentrating mechanisms (CCMs) to cope with low environmental concentrations of carbon dioxide [12]. This mechanism has been previously identified for certain SLC4 family transporters in marine diatoms [81]. Diatom CCMs also include CAs [82]. CAs are also typically involved in pH homeostasis to accelerate the hydration/dehydration reactions in the CO_2_/HCO_3_^−^ buffering system [80]. Further studies are required to precisely characterize the great diversity of CA types, subcellular locations, and functions found across the 12 studied strains, which agrees with the previously described large variety of CA across diatoms [82]. In particular, the frequent enhancement of LCIB/C βCA domain-containing proteins at acidic pH suggests that this enzyme may play a role in adaptation to low pH in diatoms. Lastly, to our knowledge, there is limited information on the function of the observed widespread enrichment of silicic acid transporters at acidic pH. This response might be associated with larger frustule-bound silica quotas and reduced valve porosity [83].

Identifying pH-responsive plastic genes represents a first step toward elucidating their role in diatom mechanisms for acid and alkaline tolerance, which is a significant finding, given the considerable proportion of unannotated proteins identified in the examined transcriptomes. Further studies should explore the functional role and the adaptive potential of identified plastic responses and exclusive molecular mechanisms; e.g. by assessing species tolerance to extreme pH and establishing the precise correlation between gene expression and maximum tolerance [84].

## Supplementary Material

Supplementary_material_final_ycaf078

Supplementary_Table_S4_ycaf078

Supplementary_Table_S5_078

## Data Availability

The raw sequencing data for this study have been deposited in the European Nucleotide Archive (ENA) at EMBL-EBI under accession number PRJEB76501 (https://www.ebi.ac.uk/ena/browser/view/PRJEB76501). The datasets supporting the results of this study, including *de novo* transcriptomes, predicted protein sequences, functional annotations, gene expression data, differential expression analysis, and enrichment analyses, have been deposited in Zenodo under DOI https://doi.org/10.5281/zenodo.14926660. Additional data are available from the corresponding author upon request.
