# Supplementary material for: Molecular complexity of the differential growth of freshwater diatoms along pH gradients
Source: ISME Commun. 2025 May 6;5(1):ycaf078. doi: 10.1093/ismeco/ycaf078 (PMC12145874; doi:10.1093/ismeco/ycaf078)
Supplement: Supplementary_material_final_ycaf078 [file supplementary_material_final_ycaf078.pdf]

Supplementary Information

Molecular complexity of the differential growth of freshwater diatoms along pH gradients

Running head: Complex molecular pH response in diatoms

Xènia Rodríguez-Miret[1], Marisol Felip[2,1], Eric Pelletier[3,4], Jordi Catalan[5,1]

[1]Centre de Recerca Ecològica i Aplicacions Forestals, CREAF, 08193 Cerdanyola del Vallès, Spain

[2]Departament de Biologia Evolutiva, Ecologia i Ciències Ambientals, Universitat de Barcelona, UB, 08028 Barcelona, Spain

[3]Génomique Métabolique, Genoscope, Institut de Biologie François Jacob, CEA, CNRS, Univ. Evry, Université Paris-Saclay, 91000 Evry, France

[4]Research Federation for the Study of Global Ocean Systems Ecology and Evolution, FR2022/Tara Oceans GO-SEE, CNRS, 75016 Paris, France

[5]Consejo Superior de Investigaciones Científicas, CSIC, 08193 Cerdanyola del Vallès, Spain

Corresponding author: Xènia Rodríguez Miret

Centre de Recerca Ecològica i Aplicacions Forestals (CREAF)

Campus de Bellaterra (UAB), Edifici C

08193 Cerdanyola del Vallès

Barcelona, Spain

Email: x.rodriguez.miret@gmail.com

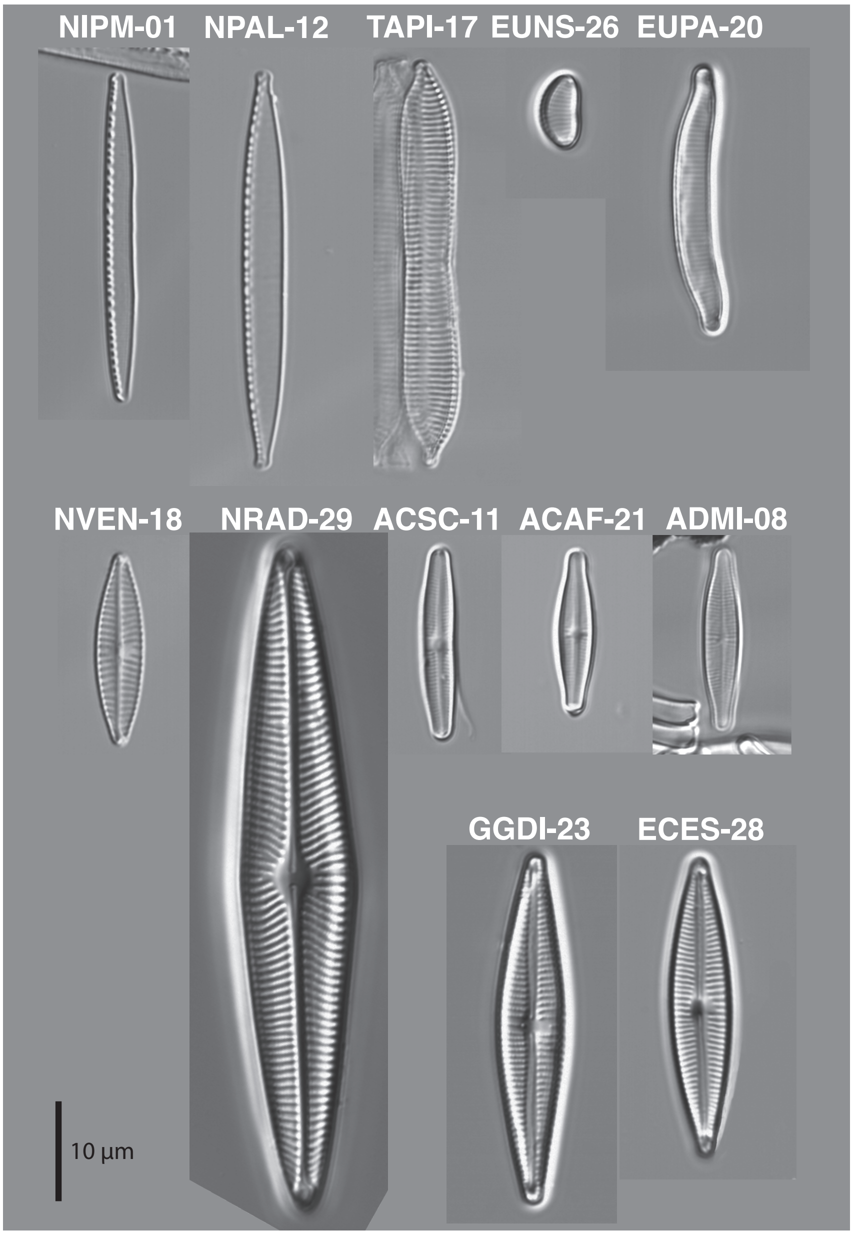

Figure S1. Images of the twelve diatom strains included in this study. The information about the taxonomic classification and the lake of origin for each strain is provided in Supplementary Table S2. All images are scaled to the same size for direct comparison. A scale bar representing 10 μm is included for reference.

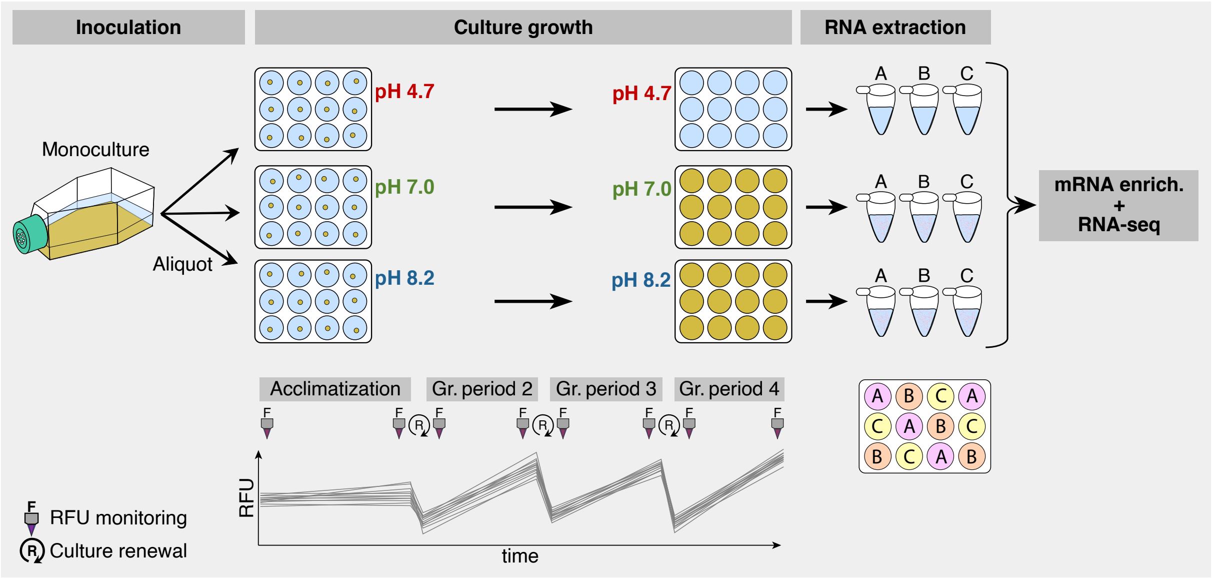

Figure S2. Common garden experiment design. This process was performed for each of the twelve studied diatom strains. Yellow-brownish surfaces represent diatom biofilms. The growth and relative fluorescence units (RFU) pattern shown here is an example that illustrates the process clearly. Culture renewal can refer to either growth medium renewal or full reinoculation, depending on the culture growth phase. The plate annotated with letters A, B, and C indicates how culture samples from wells were pooled at the end of the experiment into the replicates used for RNA extraction and sequencing.

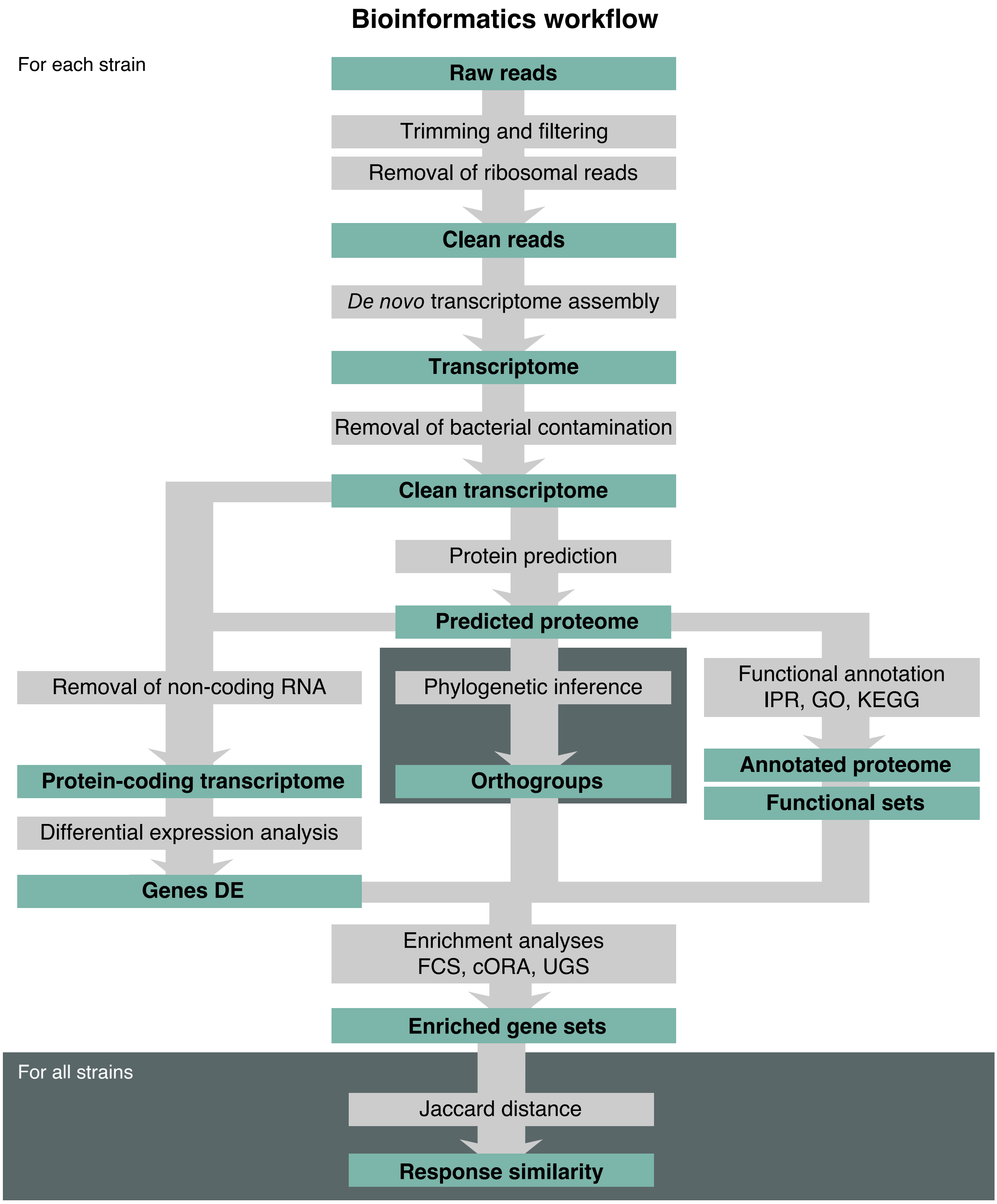

Figure S3. Bioinformatics workflow. Processes shown on a white background were performed for each strain independently, whereas those shown on a dark turquoise background involved all strains.

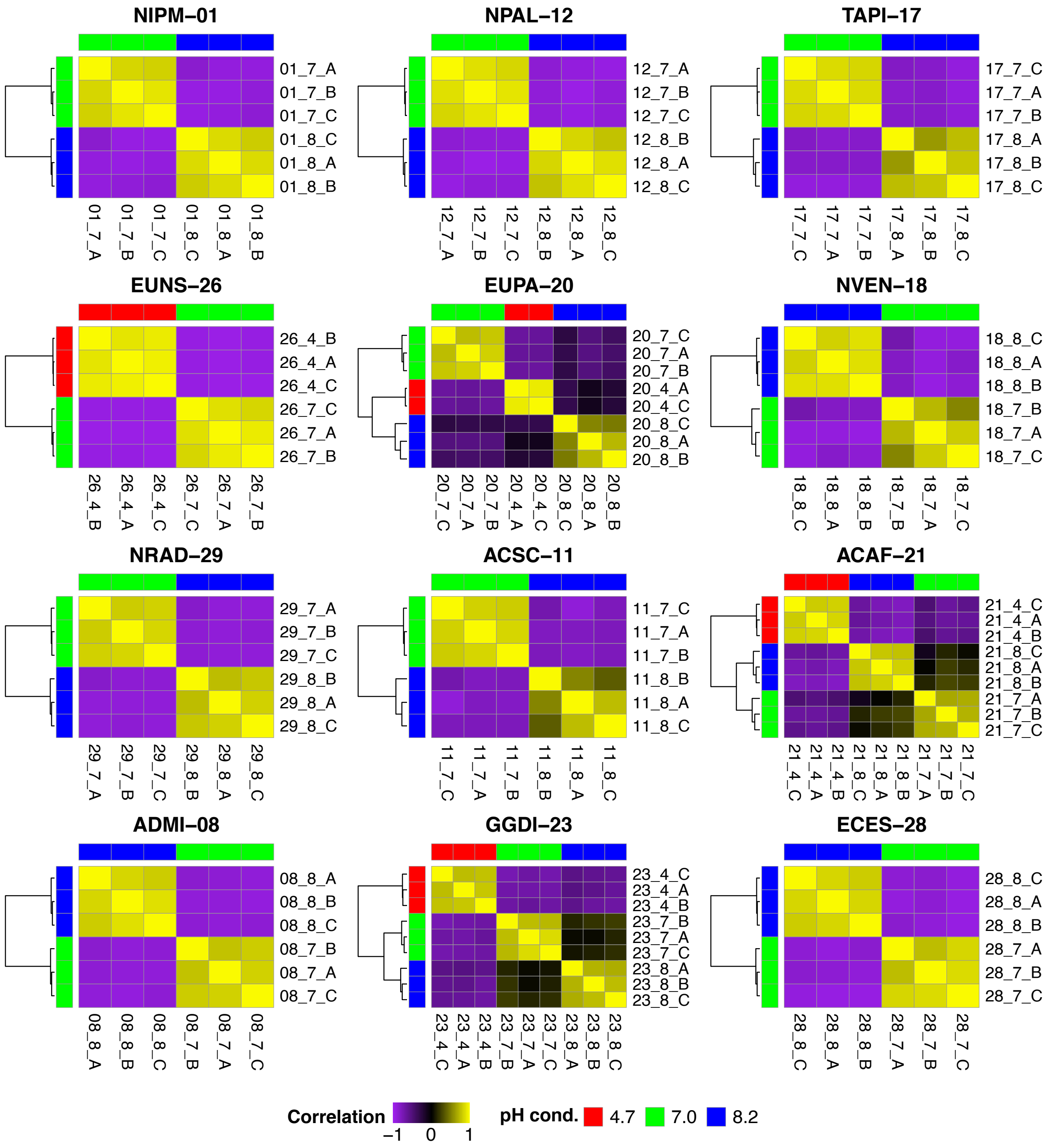

Figure S4. Sample-to-sample gene expression correlation within diatom strains. Gene expression values were log2-transformed and centered, and Pearson correlation coefficients were calculated between samples. Euclidean distances between samples were used to generate the dendrograms for sample ordering along the axes (shown only on the y-axis). Samples were named using the strain number followed by the truncated pH condition (4, 7, or 8) and the replicate (A, B, or C).

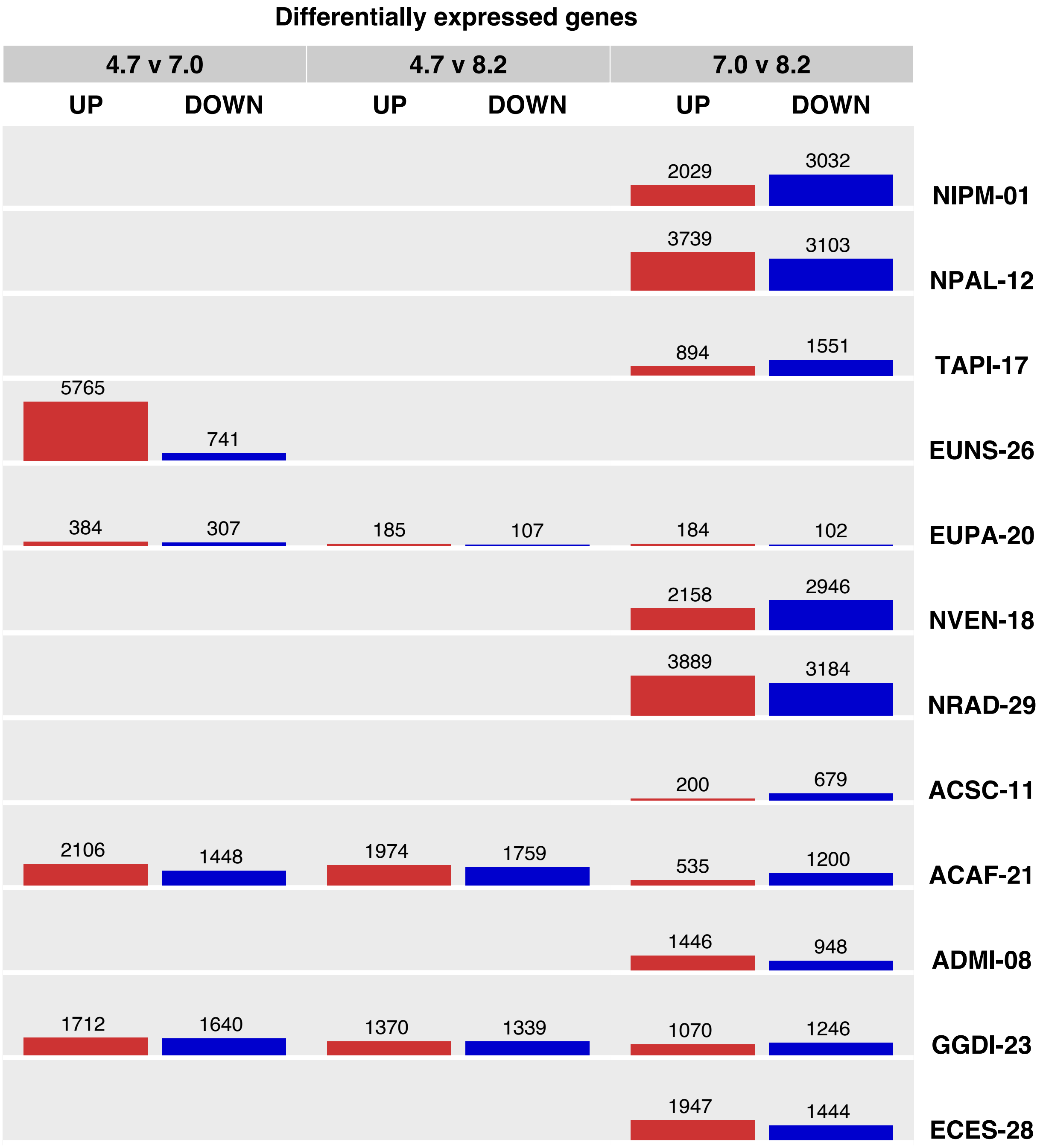

Figure S5. Number of differentially expressed genes in each pairwise pH comparison for the twelve diatom strains. For each contrast, genes were considered differentially expressed if FDR $\leq$ 0.01. In each comparison, expression at the lowest pH was compared to that at the highest. Note that comparisons including pH 4.7 for acid-intolerant strains could not be performed due to population collapse.

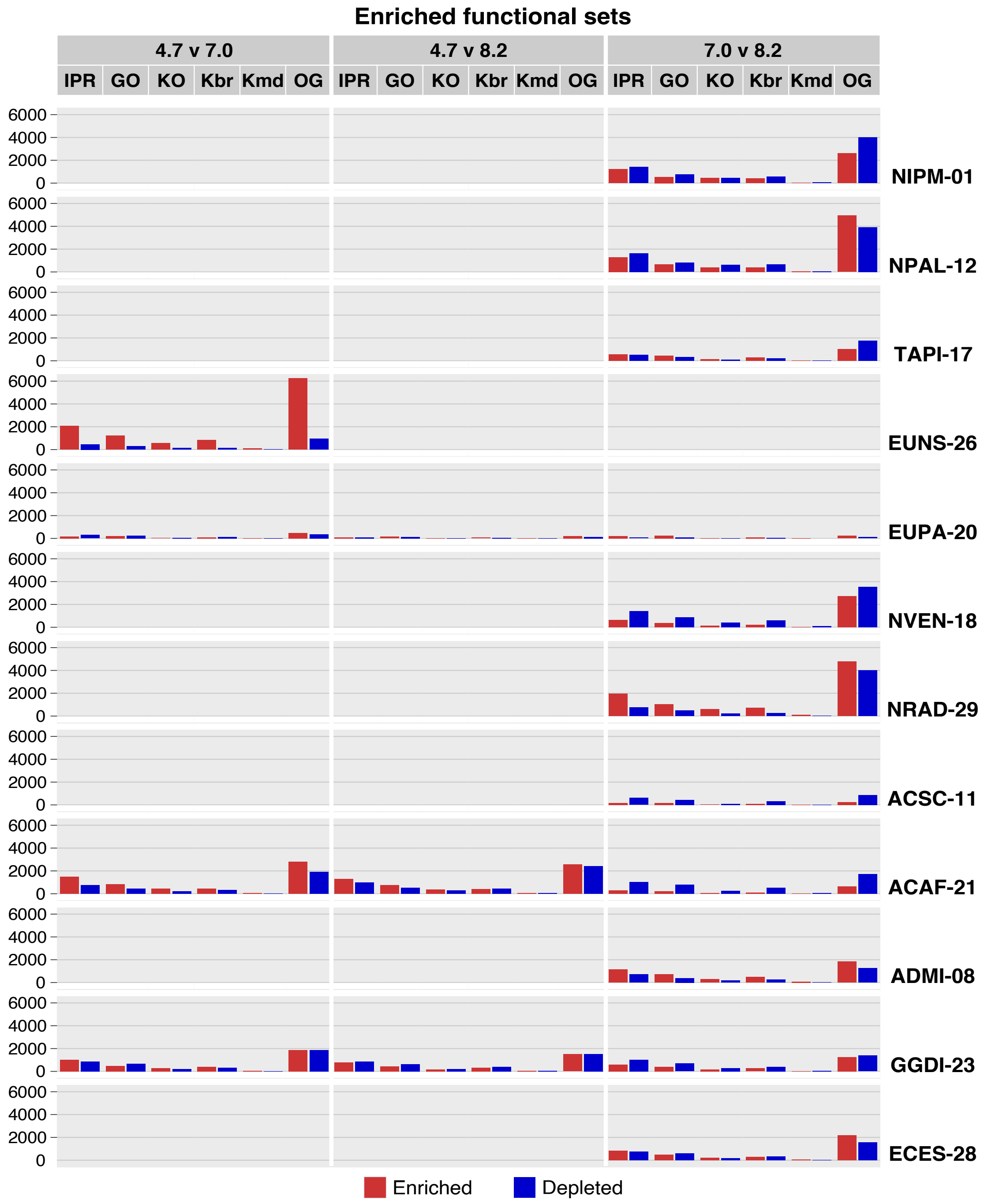

Figure S6. Number of enriched and depleted gene sets per pH comparison in the twelve diatom strains. For each contrast, sets were considered enriched or depleted if FDR $\leq$ 0.01. A gene set was considered enriched if the enrichment was significant in at least one of the three methods (FCS, ORA, and UGS), and similar for depletions. Note that comparisons including pH 4.7 for acid-intolerant strains could not be performed due to population collapse. IPR, InterPro; GO, Gene Ontology; KO, KEGG Orthology; Kbr, KEGG BRITE; Kmd, KEGG MODULE; OG, orthogroup.

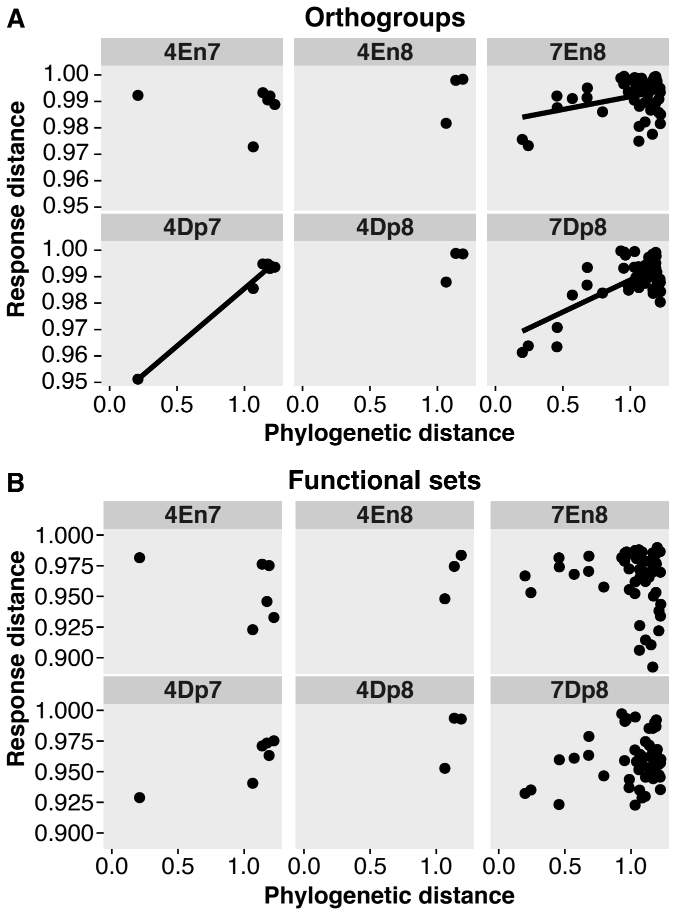

Figure S7. Comparison between phylogenetic and gene set response distances among strains. Each data point represents a contrast between two strain-pH enrichment groups. The x-axis represents the phylogenetic distance between the two strains of the two compared groups, retrieved from the phylogenetic tree obtained with OrthoFinder (Figure 3). The y-axis represents the gene set response distance between the two compared groups (Figure 8). Only pairwise contrasts between groups with the same enrichment direction and pH comparison were considered, and these were classified into orthogroup (A) and functional sets (B) facets. For instance, the contrast between orthogroups in NPAL-12_7En8 and NIPM-01_7En8 groups is represented by one point in the "Orthogroups 7En8" facet. See Materials and Methods for more details. Trend lines are shown for significant linear correlations (p-value $\leq$ 0.01) in each facet. Note that the y-axes do not start at zero.

Table S1. Nutrient concentrations in WC and PM media. All concentrations are shown in mg L^{-1}.

	WC medium	PM medium	PM to WC
**Macronutrients**			
$CaCl_2 \cdot 2\,H_2O$	36.76	0.37	1/100
K_2HPO_4	8.71	2.90	1/3
$MgSO_4 \cdot 7\,H_2O$	36.97	3.70	1/10
$Na_2SiO_3 \cdot 9\,H_2O$	28.42	14.21	1/2
$NaHCO_3$	12.60	3.15	1/4
$NaNO_3$	85.01	56.70	2/3
**Micronutrients**			
$Na_2 \cdot EDTA$	4.36	2.18	1/2
$FeCl_3 \cdot 6\,H_2O$	3.15	1.58	1/2
$CuSO_4 \cdot 5\,H_2O$	0.01	0.005	1/2
$MnCl_2 \cdot 4\,H_2O$	0.18	0.09	1/2
$ZnSO_4 \cdot 7\,H_2O$	0.022	0.011	1/2
$CoCl_2 \cdot 6\,H_2O$	0.01	0.005	1/2
$Na_2MoO_4 \cdot 2\,H_2O$	0.006	0.003	1/2
H_3BO_3	1.00	0.50	1/2
**Vitamins**			
B_1	1.00×10^{-1}	5.00×10^{-2}	1/2
H	5.00×10^{-4}	2.50×10^{-4}	1/2
B_{12}	5.00×10^{-4}	2.50×10^{-4}	1/2

Table S2. Taxonomic classification and lake of origin of the twelve diatom strains. Strain names consist of a common species abbreviation followed by the laboratory identification number. Measured pH values during sampling: Lake Aixeus, 4.89; Lake Redon (Conangles), 6.97; Lake Redó (Aigüestortes), 7.04; Lake Estanya, 8.11.

**Strain name**	**Lake**	**Taxonomy**
		Bacillariophyceae
		Bacillariales
		Bacillariaceae
NIPM-01	Redó	*Nitzschia perminuta* Grunow
NPAL-12	Estanya	*Nitzschia palea* (Kützing) W.Smith
TAPI-17	Estanya	*Tryblionella apiculata* W.Gregory
		Eunotiales
		Eunotiaceae
EUNS-26	Aixeus	*Eunotia* sp.
EUPA-20	Aixeus	*Eunotia paludosa* Grunow
		Naviculales
		Naviculaceae
NVEN-18	Estanya	*Navicula veneta* Kützing
NRAD-29	Estanya	*Navicula radiosa* Kützing
		Achnanthales
		Achnanthidiaceae
ACSC-11	Estanya	*Achnanthidium* aff. *sehuencoense* E.Morales
ACAF-21	Redon	*Achnanthidium affine* (Grunow) Czarnecki
ADMI-08	Estanya	*Achnanthidium minutissimum* (Kützing) Czarnecki *s.s.*
		Cymbellales
		Gomphonemataceae
GGDI-23	Redon	*Gomphonema graciledictum* E.Reichardt
		Cymbellaceae
ECES-28	Estanya	*Encyonopsis cesatii* (Rabenhorst) Krammer

Table S3. RNA concentration in sample replicates of the twelve diatom strains under the three pH conditions used in the common garden experiment. For each replicate, 20–50 µl of RNA extract was sent for sequencing.

		Concentration (in ng µl^{-1})		
**Strain**	**Replicate**	**pH 4.7**	**pH 7.0**	**pH 8.2**
NIPM-01	A	-	37.2	62.0
	B	-	38.4	54.4
	C	-	29.8	64.6
NPAL-12	A	-	41.0	52.0
	B	-	36.2	93.6
	C	-	48.2	45.8
TAPI-17	A	-	33.0	30.4
	B	-	45.0	44.2
	C	-	61.2	48.2
EUNS-26	A	11.3	6.0	-
	B	17.7	4.2	-
	C	11.2	5.2	-
EUPA-20	A	6.4	8.0	12.5
	B	-	5.7	12.2
	C	16.4	5.0	7.8
NVEN-18	A	-	20.8	70.2
	B	-	24.0	41.2
	C	-	28.4	51.2
NRAD-29	A	-	27.2	77.6
	B	-	30.0	75.2
	C	-	28.8	88.0
ACSC-11	A	-	16.8	18.8
	B	-	11.2	36.4
	C	-	20.0	29.8
ACAF-21	A	26.0	25.0	33.4
	B	19.4	22.4	17.7
	C	30.8	22.0	24.2
ADMI-08	A	-	23.2	38.0
	B	-	22.2	39.8
	C	-	18.4	35.4
GGDI-23	A	13.7	10.3	26.4
	B	10.3	11.1	21.2
	C	16.4	10.3	23.4
ECES-28	A	-	5.2	13.8
	B	-	5.9	15.6
	C	-	5.1	14.1
